# Curcumin Down-Regulates DNA Methyltransferase 1 and Plays an Anti-Leukemic Role in Acute Myeloid Leukemia

**DOI:** 10.1371/journal.pone.0055934

**Published:** 2013-02-13

**Authors:** Jianhua Yu, Yong Peng, Lai-Chu Wu, Zhiliang Xie, Youcai Deng, Tiffany Hughes, Shun He, XiaoKui Mo, Ming Chiu, Qi-En Wang, Xiaoming He, Shujun Liu, Michael R. Grever, Kenneth K. Chan, Zhongfa Liu

**Affiliations:** 1 Division of Hematology, Department of Internal Medicine, College of Medicine, The Ohio State University, Columbus, Ohio, United States of America; 2 Comprehensive Cancer Center, The Ohio State University, Columbus, Ohio, United States of America; 3 Department of Molecular and Cellular Biochemistry, The Ohio State University, Columbus, Ohio, United States of America; 4 Division of Pharmaceutics, College of Pharmacy, The Ohio State University, Columbus, Ohio, United States of America; 5 College of Pharmacy, Third Military Medical University, Chongqing, China; 6 Center for Biostatistics, The Ohio State University, Columbus, Ohio, United States of America; 7 Department of Biomedical Engineering, The Ohio State University, Columbus, Ohio, United States of America; 8 The Hormel Institute, University of Minnesota, Austin, Minnesota, United States of America; University of Manitoba, Canada

## Abstract

Bioactive components from dietary supplements such as curcumin may represent attractive agents for cancer prevention or treatment. DNA methylation plays a critical role in acute myeloid leukemia (AML) development, and presents an excellent target for treatment of this disease. However, it remains largely unknown how curcumin, a component of the popular Indian spice turmeric, plays a role in DNA hypomethylation to reactivate silenced tumor suppressor genes and to present a potential treatment option for AML. Here we show that curcumin down-regulates DNMT1 expression in AML cell lines, both *in vitro* and *in vivo*, and in primary AML cells *ex vivo*. Mechanistically, curcumin reduced the expression of positive regulators of DNMT1, p65 and Sp1, which correlated with a reduction in binding of these transcription factors to the DNMT1 promoter in AML cell lines. This curcumin-mediated down-regulation of DNMT1 expression was concomitant with *p15^INK4B^* tumor suppressor gene reactivation, hypomethylation of the *p15^INK4B^* promoter, G1 cell cycle arrest, and induction of tumor cell apoptosis in vitro. In mice implanted with the human AML MV4–11 cell line, administration of curcumin resulted in remarkable suppression of AML tumor growth. Collectively, our data indicate that curcumin shows promise as a potential treatment for AML, and our findings provide a basis for future studies to test the clinical efficacy of curcumin – whether used as a single agent or as an adjuvant – for AML treatment.

## Introduction

In conjunction with post-translational histone modifications (including acetylation and methylation), DNA methylation at cytosine bases found within 5′-cytosine-phospho-guanosine (CpG) sequences in gene promoter regions represents an epigenetic mechanism that controls gene transcription, genome stability and genetic imprinting. DNA methyltransferase 1 (DNMT1), which catalyzes the transfer of methyl groups to DNA, represents a crucial mediator of DNA methylation. In a variety of solid tumors and blood cancers, aberrant hypermethylation of CpG-rich regions (>55% CG content, 0.5-4 kb in length, the so-called “CpG islands”) in the promoters of tumor suppressor genes (TSGs) results in their transcriptional silencing [1], [2].

Preclinical and clinical studies have both demonstrated that DNA methylation inhibitors, including decitabine and 5-azacytidine – which are both the Food and Drug Administration (FDA)-approved azanucleoside drugs – are effective treatments for hematological malignancies. These agents have been reported to suppress tumor growth by reversing aberrantly hypermethylation in the promoters of inactivated TSGs (e.g. *p15^INK4B^)*, allowing re-expression of TSGs, thereby restoring normal cell cycle regulation, proliferation, apoptosis, and differentiation [3], [4]. Though azanucleosides are effective treatment for some malignancies, these drugs are somewhat limited in their utility due to their relative nonspecificity, and the undesirable side effects (i.e. myelosuppression) they can cause; furthermore, cancer cells can rapidly become chemo-resistant to these drugs; moreover, since azanucleoside drugs are only active during the S phase of the cell cycle, they have limited efficacy as treatments for malignancies characterized by hypoproliferative cancer cells [5]. Thus, the discovery and development of novel DNA methylation inhibitors that are effective, but less toxic, are needed [6]. Several non-nucleoside DNA methylation inhibitors, such as procainamide [7] and RG108 [8], have been identified; however, none of these drugs possess hypomethylating activity comparable to that of azanucleosides [9].

Curcumin, a major yellow pigment extracted from turmeric – which is widely consumed as a dietary additive to enhance coloring and flavoring in a variety of food [10] – has been long used as a treatment for inflammation, skin wounds, cough, as well as certain tumors [11], [12]. A variety of molecular mechanisms have been proposed to mediate these effects, and other groups have reported that curcumin acts as a scavenger of free radicals [13], an inhibitor of NF-κB nuclear translocation [14], and a modulator of histone deacetylase (HDAC) and histone acetyltransferase (HAT) [15], [16], [17]. Notably, in the MDA-MB-231 cell line, long-term treatment with low doses of curcumin also alters the expression of TSGs, such as *E-cadherin-11* [18]. This finding is consistent with our recent computer modeling study, which showed that curcumin and its analogs may inhibit enzymatic activity of DNA methyltransferase, with an IC_50_ of 30 nM [19]. However, the means by which curcumin induces DNA hypomethylation, and whether this hypomethylating activity is associated with anti-tumor activity - especially in AML - remain largely unknown.

NF-κB is a ubiquitous transcription factor with pleiotropic effects on cell growth, survival, tumorigenesis, and a variety of other cellular activities [20]. We have recently demonstrated that NF-κB forms a complex with Sp1 and binds to the promoter of *DNMT1*, resulting in *DNMT1* transactivation in the MV4–11 AML cell line [21]. However, whether curcumin modulates this positive regulation of DNMT1, and in turn controls DNMT1 expression during AML, remains to be determined.

In this study, we found that curcumin down-regulated DNMT1 expression in AML cells. This occurred, at least in part, through down-modulation of two positive regulators of DNMT1: Sp1 and the NF-κB component, p65. We also found that curcumin-mediated down-regulation of DNMT1 was associated with reactivation of TSGs and tumor suppression, both *in vivo* and *in vitro*.

## Materials and Methods

### Materials

Curcumin, methanol, acetonitrile (HPLC grade), ammonium formate, ammonium acetate, ammonium bicarbonate, 5-methyl-2-deoxycytidine (5mdC), 2-deoxycytidine (2dC), 2-deoxyguanosine (2dG), nucleophosphatase (NP1), snake venom phosphatase (SVP), alkaline phosphatase (AP), deoxynucleotide triphosphate (2.5 mM), AmpliTaqGold polymerase, and 10X PCR buffer were purchased from Sigma-Aldrich (St. Louis, MO). Decitabine was obtained from the National Cancer Institute and used without further purification. The primers for amplification of *p15^INK4B^* and its bisulfite-converted promoter region, and also for *DNMT1* and its Sp1-binding promoter region, were purchased from Sigma-Aldrich or Integrated DNA Technology (IDT, Coralville, IA). M. SssI methylase, s-adenosylmethionine (SAM) (3.2 mM) and 10X incubation buffer were purchased from New England Biolabs (Ipswich, MA). DNMT1 antibody was purchased from New England Biolabs, β–actin antibody from Aldrich-Sigma, and GAPDH antibody (HRP Conjugate) from Cell Signaling Technology (Danvers, MA). Sp1, NF-κB p65, and Histone 2B (H2B) antibodies were purchased from Santa Cruz Biotech (Santa Cruz, CA) and cleaved caspase-3 (Asp175) and cleaved caspase-9 (Asp315) antibodies from Cell Signaling Technology.

### Cytotoxicity and Cell Cycle Analysis

The leukemia cell lines, K562 (erythroleukemic cell line), MV4–11 (AML), HL-60 [acute promyelocytic leukemia (APL), a subtype of AML], ML-1 (AML), Kasumi-1 (AML), and THP-1 (AML) were purchased from ATCC (Manassas, VA) and cultured at 37°C in an incubator under 5% CO_2_ atmosphere in RPMI media (VWR International, West Chester, PA) supplemented with fetal bovine serum (Life Technologies, Carlsbad, CA) (20% for Kasumi-1 and 10% for the other cell lines) and 1% (v/v) penicillin/streptomycin (Life Technologies) antibiotic solution. For the *ex vivo* studies, mononuclear cells were isolated from bone marrow (BM) of patients with AML (from the Leukemia Tissue Bank at The Ohio State University) by Ficoll-Hypaque (Nygaard, Oslo, Norway) gradient centrifugation, and then were cultured in serum-free expansion medium (SFEM) (StemCell technologies, Vancouver, Canada) supplemented with granulocyte-macrophage colony-stimulating factor (GM-CSF, 50 ng/ml), interleukin (IL)-3 (20 ng/ml), IL-6 (20 ng/ml) and stem cell factor (SCF, 100 g/ml) (All were purchased from R&D Systems, Minneapolis, MN). These cells were treated with indicated concentrations of curcumin or decitabine (as a positive control) for time periods as indicated. Cell cycle analysis of MV4–11 cells was performed via flow cytometry using a FACSCalibur (Beckman Coulter, Fullerton, CA). Aforementioned human AML samples were obtained from patients who gave informed consent. The study protocol was conducted in accordance with the Declaration of Helsinki and was approved by The Ohio State University Institutional Review Boards.

### Cell Lysis and Immunoblotting

Cells or tumor tissues were homogenized and lysed in ice-cold lysis buffer (20 mM HEPES, pH 7.0, 150 mM NaCl, 0.1% NP40 supplemented with 1 mM β-glycerophosphate, 1 mM Na_3_VO_4_, 1 mM NaF, 1 mM benzamide and 1 mM phenylmethylsulfonyl fluoride) with protease inhibitors (Protease Inhibitor Cocktail Set III, Calbiochem-Novabiochem Corporation, La Jolla, CA), incubated on ice for 20 min, then centrifuged at 12,000 g for 10 min at 4°C. Proteins in the supernatants were resolved on 4–15% SDS-polyacrylamide gradient gels (Bio-Rad Laboratories, Hercules, CA), then transferred onto nitrocellulose membranes, incubated with appropriate antibodies, and signals were detected using ECL reagents (GE Healthcare Bio-Sciences Corp., Piscataway, NJ).

### Quantitative Real-Time RT-PCR Assays

Quantitative RT–PCR was used to assess *DNMT1* and *p15^INK4B^* mRNA expression levels. Total RNA was extracted with Trizol reagent (Life Technologies) and reverse transcribed by reverse transcriptase (Life Technologies). Quantitative real-time RT-PCR reactions were performed in triplicate using Taqman gene expression assay (Life Technologies) with an ABI prism 7700 detector (Life Technologies). The target gene expression values were normalized to *GAPDH* internal control, and are reported here as fold change (x 2^(−ΔΔCt)^) relative to non-treated samples.

### Electrophoretic Mobility-Shift Assays (EMSAs) and Antibody-Supershift Assays

Nuclear extracts were prepared using NE-PER (Nuclear and Cytoplasmic Extraction Reagent; Pierce, Rockford, IL). EMSA was performed as previously described [21], using nuclear extracts and ^32^p-labeled oligonucleotides containing two Sp1 binding sites on the *DNMT1* promoter. For antibody gel supershift assay, nuclear extracts were pre-incubated with Sp1 antibodies at 4°C for 16 h before adding the probes. The oligonucleotides and their complementary sequences were chemically synthesized, annealed, and labeled with ^32^P-dCTP by Klenow fragment (Life Technologies).

### DNA Methylation Analysis of the *p15^ INK4B^* Promoter Region

Genomic DNA was isolated from MV4–11 cells, treated with bisulfite using the Epi methylation Kit (Qiagen, Minneapolis, MN) according to manufacturer’s instructions, then amplified via PCR. The sequences of *p15^ INK4B^* primers were: forward 5′-GGG AGG GTA ATG AAG TTG AGT TTA-3′, and reverse 5′- ACC CTA AAA CCC CAA CTA CCT AAA T -3′. PCR amplifications were performed as previously described [22].

### *In Vitro* Cell Proliferation

MV4–11 cells were seeded in a 96-well plate (10,000 cells per well), then treated with vehicle (DMSO) or curcumin at varied concentrations. CellTiter 96 AQueous One Solution Reagent (Promega, Madison, WI) was used according to manufacturer’s instructions to determine cell growth capacity. Briefly, after CellTiter 96 AQueous One Solution Reagent was added at the indicated time points, cells were incubated at 37°C for an additional 4 h, and a spectrophotometer was used to measure absorbance at 490 nm.

### Xenograft Animal Model

Female athymic nu/nu mice (4–6 weeks old, 18–22 g) were obtained from Charles River Laboratory (Wilmington, MA). Animals were given sterile rodent chow and water *ad libitum* and were housed in sterile filter-top cages with 12 h light/dark cycles. All experiments were reviewed and approved by The Ohio State University Institutional Laboratory Animal Care and Use Committee. MV4–11 cells (5×10^6^ cells per mouse) were suspended in 50% Matrigel (Becton Dickinson, Franklin Lakes, NJ) and subcutaneously implanted into both right and left flanks of the athymic nu/nu mice. When tumors measuring 100 to 200 mm^3^ had grown, treatments were initiated. Mice were randomly assigned into two cohorts with 6 mice per group for the anti-tumor growth activity studies. Curcumin was given intraperitoneally as a solution of DMSO: ethanol: saline (10∶3:7), at a dose of 100 mg/kg daily, five days per week, for 4 weeks. The placebo formulation was used as a control. Tumor volume was calculated by using the equation: V = 2×A×B^2^/3, where A is the longer (horizontal as width) diameter (mm) and B is the shorter (perpendicular as depth) diameter (mm) and expressed in cubic millimeters. After 4 weeks, mice were euthanized and tumor tissues were excised, weighed, snap frozen in liquid nitrogen, and stored in a −80°C freezer until analysis.

## Results

### Curcumin Down-regulates DNMT1 Expression in AML Cells *in vitro* and *ex vivo*

**Figure 1:**
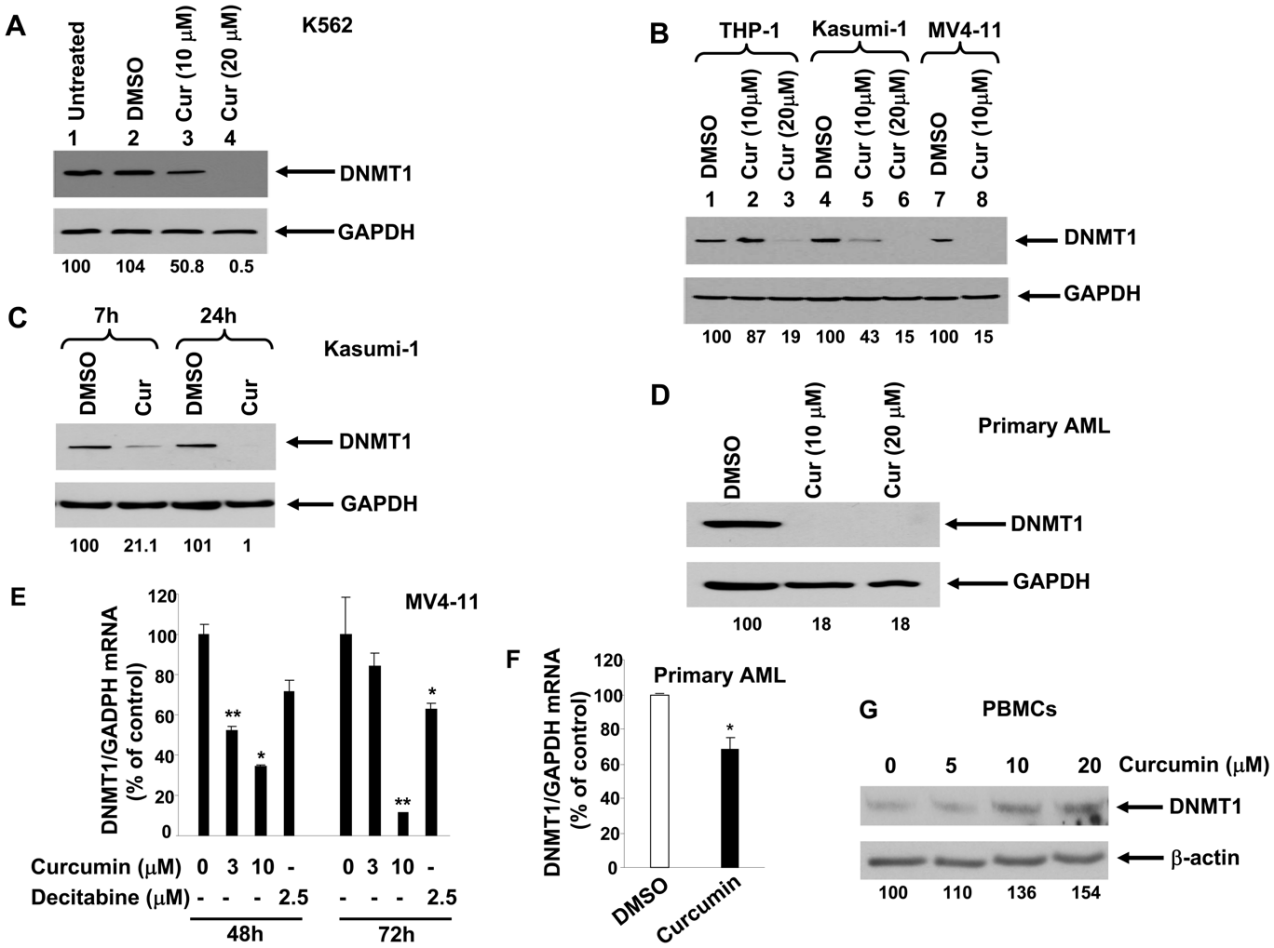
Curcumin down-regulates DNMT1 mRNA and protein expression in myeloid leukemia cells *in vitro* and *in vivo*. (A–D) DNMT1 and GAPDH antibodies were used for immunoblotting of total cell lysates from K562 (A), THP-1 (B), Kasumi-1 (B and C), and MV4–11 cells (B), or primary AML cells (D). These cells were either left untreated, treated with vehicle (DMSO), or treated with 10 µM curcumin (“Cur”) or 20 µM curcumin. Treatment lasted for 24 h (A, B), 7 and 24 h (C), or 72 h (D). Data shown in (A–D) are from one representative experiment of three total experiments, each with similar data. Numbers beneath each lane represent densitometric quantification of DNMT1, normalized to GAPDH. Values, depicted as percent change, were calculated as change relative to incubation with carrier control. (E, F) MV4–11 cells (E) or primary AML cells (F) were incubated with carrier containing either curcumin (curcumin was used at the concentrations indicated for MV4–11 cells, and 10 µM for primary cells) or decitabine (2.5 µM; positive control). DNMT1 transcript was assessed by RT PCR, and normalized to GAPDH internal control. *, *p*<0.05; **, *p*<0.01. (G) Peripheral blood mononuclear cells (PBMCs) were treated with the indicated concentrations of curcumin for 24 h. Total cell lysates were then used for immunoblot assay to detect DNMT1 and β-actin. Results from one of three donors with similar data are shown. Numbers beneath each lane represent densitometric quantification of DNMT1, normalized to β-actin.

Our previous data demonstrate that curcumin can cause hypomethylation via blockade of the catalytic thiol group of DNMT1 [23]. Here, we determined whether curcumin was able to induce hypomethylation in myeloid leukemia cells by down-regulating DNMT1 gene expression. For this purpose, we treated the K562 myeloid leukemia cell line with curcumin, used at a concentration of either 10 µM or 20 µM. Immunoblotting indicated that curcumin indeed down-regulated DNMT1 protein expression level in a dose-dependent fashion (Figure 1A). Consistent with data obtained in the K562 cell line, curcumin also down-regulated DNMT1 protein expression in the AML cell lines: THP-1, Kasumi-1, and MV4–11 (Figure 1B). Observations in two additional AML cell lines, ML-1 and HL-60, were similar (data not shown). Next, we again used AML cell lines to demonstrate the time-dependent manner in which this curcumin-mediated inhibition of DNMT1 protein expression occurs (Figure 1C). We observed similar patterns of curcumin-mediated inhibition of DNMT1 expression in AML cells from patient samples, thus confirming our findings in primary cells (Figure 1D). Our data also demonstrated that inhibition of DNMT1 occurred at the level of mRNA expression in the MV4–11 cell line, as well as primary AML cells (Figure 1E and 1F). However, we did not observe DNMT1 downregulation after treating normal peripheral blood mononuclear cells (PBMCs) with curcumin (Figure 1G), thus suggesting that curcumin may selectively downregulate DNMT1 expression in tumor cells, but not in normal cells.

### Curcumin Inhibits Expression of p65 and Sp1 and their Association with the *DNMT1* Promoter

**Figure 2:**
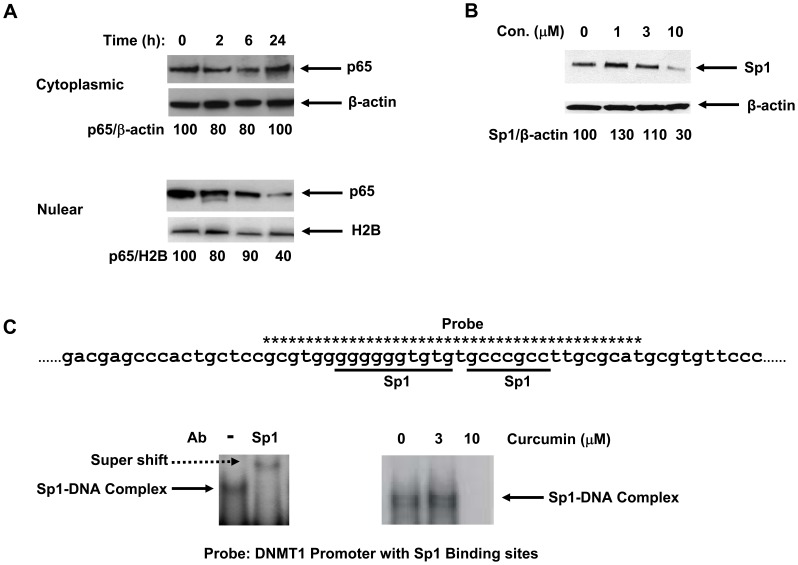
Curcumin inhibits p65 and Sp1 protein expression and the association of Sp1 with the *DNMT1* promoter. (**A**) After MV4–11 cells were treated with curcumin (10 µM) for the indicated periods of time, cytoplasmic and nuclear extracts were analyzed for p65 protein expression via immunoblotting. Numbers beneath each lane represent densitometric quantification of p65 protein expression, following normalization to β-actin (cytoplasmic) or H2B (nuclear) expression. (B) Whole cell lysates were prepared from MV4–11 cells incubated for 24 h with carrier alone or carrier containing curcumin (concentrations as indicated). Sp1 protein expression was assessed via immunoblotting, quantified via densitometry, and normalized to β-actin loading control. Data shown in (A) and (B) are from one representative experiment of three total experiments, each with similar data. (C) A ^32^P-labeled probe, containing two Sp1 binding sites on the *DNMT1* promoter (top panel) and an Sp1 antibody were used to perform EMSA assays using nuclear extracts from MV4–11 cells, either before treatment (bottom, left panel) or following incubation for 72 h or 48 h (not shown) with carrier alone or carrier containing the indicated concentration of curcumin (bottom, right panel).

Our previous study indicated that DNMT1 expression is positively regulated by Sp1 and the NF-κB signaling component, p65, which physically interact and bind to the *DNMT1* promoter [21]. This finding prompted us to investigate whether curcumin can inhibit protein expression of both transcription factors in AML cells. For this purpose, we treated the AML cell line, MV4–11, with curcumin or vehicle control. Immunoblotting indicated that a moderate curcumin-mediated down-regulation of p65 protein occurred within total cellular protein lysates (data not shown), but when cytoplasmic and nuclear protein fractions were isolated separately, we found that curcumin treatment decreased p65 protein existing within the nucleus, while cytoplasmic p65 remained unaltered (Figure 2A). Immunoblotting also showed that curcumin inhibited Sp1 expression in a dose-dependent manner in MV4–11 cells (Figure 2B). EMSA indicated that the DNMT1 promoter, which contains Sp1 binding sites, was associated with a protein complex which super-shifted with an Sp1 antibody (Figure 2C, left panel), thus indicating that Sp1 was indeed bound to the DNMT1 promoter – a finding which is consistent with our previous study [21]. Additionally, we found that curcumin treatment suppressed the physical association between the DNMT1 promoter and Sp1 or p65 (Figure 2C, right panel and data not shown, respectively).

### Curcumin Reactivates Epigenetically Silenced *p15^ INK4B^*

*p15^INK4B^* represents one of the TSGs that frequently undergoes silencing due to promoter hypermethylation in human leukemia cell lines. This can also occur in primary leukemic blasts isolated from patients with myelodysplastic syndrome (MDS) and AML [24], and hypermethylation of the *p15^INK4B^* promoter is associated with a poor prognosis in MDS and AML [24], [25], [26]. Our previous computer modeling and enzymatic study demonstrated that curcumin is capable of blocking DNMT1 enzymatic activity [19], and here we have shown that curcumin can also down-regulate DNMT1 gene expression. Therefore, we next tested whether curcumin was also able to epigenetically reactivate silenced TSGs in AML cells.

**Figure 3:**
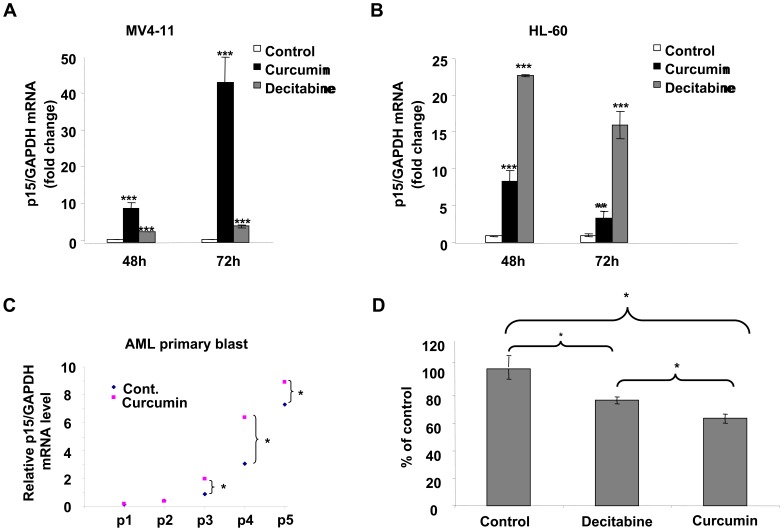
Curcumin reactivates expression of *p15^INK4B^* and causes DNA hypomethylation of its promoter in AML cells. (A, B) MV4–11 (A) or HL-60 (B) cells were treated with carrier (control), 10 µM curcumin, or 2.5 µM decitabine for the indicated periods of time. Expression of *p15^INK4B^* mRNA was then measured by quantitative real-time RT-PCR, and normalized to *GAPDH* internal control. (C) Primary leukemia cells from five patients with AML were incubated with carrier alone (control) or 10 µM curcumin for 48 h. Expression of *p15^INK4B^* mRNA was then measured by quantitative real-time RT-PCR, normalized to GAPDH internal control, and presented as relative levels. (D) MV4–11 cells were treated with carrier (control), 2.5 µM decitabine, or 10 µM curcumin for 48 h. DNA Methylation of the *p15^INK4B^* promoter was measured using a tandem LC-MS/MS method (as indicated in Materials and Methods), and is depicted relative to promoter methylation following treatment with carrier alone. *, *p*<0.05; **, *p*<0.01; ***, *p*<0.001.

**Figure 4:**
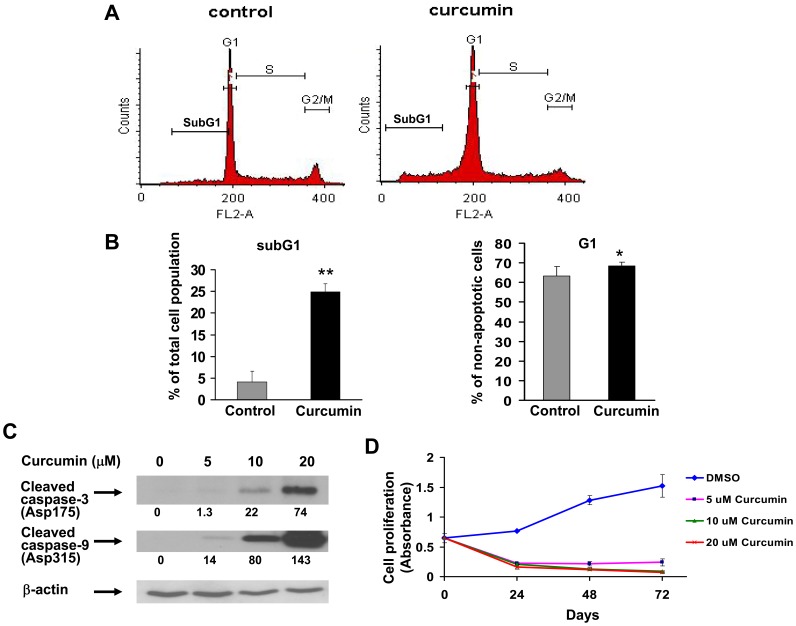
Curcumin increases the proportion of cells in SubG1 and G1 phases of the cell cycle, induces caspase cleavage, and inhibits AML cell growth *in vitro*. (A, B) MV4–11 cells were placed in serum-free medium for 48 h to synchronize cell cycle. Cells were then treated with carrier (control) or curcumin (10 µM) for 48 h, and cell cycle distribution was determined by flow cytometry. The percentages of cells in SubG1, G1, S, G2/M phases of the cell cycle were calculated for each treatment condition. Gating for each phase was performed as shown in histograms from a representative experiment in (A). Average percentages of cells in subG1 and G1 (after excluding subG1) from three independent experiments are summarized in (B). (C) MV4–11 cells were treated with curcumin for time periods as indicated. Total cell lysates were used for immunoblot assay with an antibody against cleaved caspase-3, cleaved caspase-9, or β-actin (for normalization). Results from one of three experiments with similar data are shown. Numbers beneath each lane represent densitometric quantification of cleaved caspase-3 or -9, normalized to β-actin. D) MV4–11 cells were treated with carrier alone (DMSO) or carrier containing curcumin at the indicated concentrations. After 24 h, 48 h, or 72 h, proliferation was assessed using CellTiter 96 Aqueous One Solution Reagent, and is presented here as absorbance measured by a spectrophotometer. *, *p*<0.05, **, *p*<0.01.

For this purpose, we evaluated *p15^INK4B^* expression levels in MV4–11 and HL-60 cell lines after curcumin treatment, We observed that curcumin dramatically increased *p15^INK4B^* mRNA expression in both cell lines. As shown in Figure 3A, following treatment of MV4–11 cells with 10 µM curcumin, *p15^ INK4B^* mRNA expression was increased by approximately 10- and 40-fold at 48 and 72 h, respectively. HL-60 leukemia cells behaved similarly, and also responded to curcumin by reactivating *p15^ INK4B^* expression in a time-dependent manner (Figure 3B). Most importantly, curcumin also reactivated *p15^INK4B^* expression in AML primary blast cells (Figure 4C) from 3 out of the 5 AML patients that we tested. To evaluate whether this curcumin-mediated reactivation of *p15^ INK4B^* is associated with hypomethylation of its promoter, following a 72 h treatment with 10 µM curcumin or 2.5 µM decitabine, we assessed MV4–11 cells for DNA methylation in the promoter region of the *p15^INK4B^* gene (30 CpGs, 315 bp) using a recently published LC-MS/MS method [27]. As shown in Figure 3D, *p15^INK4B^* promoter methylation in MV4–11 cells treated with curcumin or decitabine decreased by about 40% (*p = *0.017, *n* = 3) or 25% (*p = *0.038, *n* = 3), respectively.

### Curcumin Increases the Proportion of Cells in SubG1 Phase of the Cell Cycle, Induces Caspase Cleavage, and Inhibits AML Cell Growth *in vitro*

Since p15^INK4B^ is a cyclin-dependent kinase inhibitor which functions as a cell growth regulator by inhibiting cell cycle progression during the G1 phase, we next determined whether *p15^INK4B^* reactivation corresponded to changes in AML cell survival and/or proliferation. For this purpose, as shown in Figures 4A and 4B (left panel), flow cytometric analysis was conducted. After curcumin treatment, we observed a significant increase in the proportion of MV4–11 cells in subG1 phase of the cell cycle (∼24.5% with 10 µM curcumin vs 2.96% with vehicle alone), suggesting that curcumin might be inducing apoptosis of AML cells. Accordingly, immunoblotting demonstrated cleavage of caspase-3 and caspase-9 was occurring in these curcumin-treated MV4–11 cells (Figure 4C). Figure 4B (right panel) shows that treatment with curcumin resulted in a moderate – yet significant – increase in the proportion of cells in G1 phase of the cell cycle (68.3% vs. 63.4%), suggesting an additional role for curcumin in inhibiting outgrowth of AML cells by arresting the cell cycle during the G1 phase. These findings are consistent with the data in Figure 3, where we demonstrate that curcumin reactivates the cyclin-dependent kinase inhibitor, p15^INK4B^. Since curcumin treatment induced apoptosis in addition to cell cycle arrest during the G1 phase, we next investigated whether curcumin treatment might also inhibit growth of AML cells *in vitro*. As shown in Figure 4D, growth of the MV4–11 cell line was indeed inhibited by treatment with 5, 10, and 20 µM concentrations of curcumin.

### Curcumin Inhibits AML Cell Growth and Down-regulates DNMT1 Expression *in vivo*

**Figure 5:**
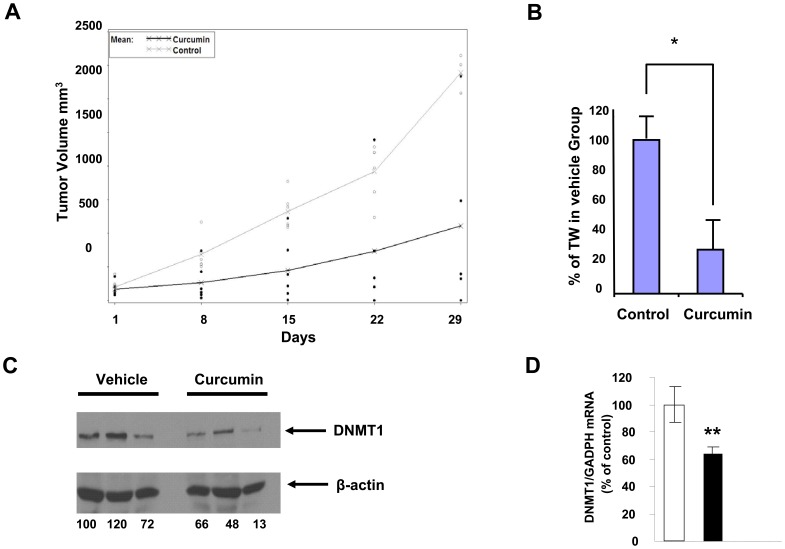
The anti-tumor effect of curcumin on MV4–11 tumor cells engrafted in nude mice. (A–D) MV4–11 cells were engrafted in nu/nu mice. Mice were then treated daily (5 days/week), with IP doses of either 100 mg/kg curcumin formulated in vehicle (DMSO, ethanol and PBS) or formulation vehicle alone (6 mice per group). (A, B) Tumor sizes were measured (as indicated in Materials and Methods) and recorded on days 1, 8, 15, 22 and 29. (A) Tumor growth in each mouse as it occurred over time is depicted here as individual dots (empty circles represent individual mice treated with carrier control, and solid dots represent individual curcumin-treated mice). Trend lines represent mean tumor size of all mice from the control group (grey) or the curcumin-treated group (black). (B) The mice described above were sacrificed on day 29 to isolate tumors. Tumors were weighed, and the average percent reduction in tumor weight observed in the curcumin-treated group was calculated relative to the average tumor weight of the mice from the vehicle control group. *, *p*<0.05. (C, D) MV4–11-engrafted tumor tissues from the mice described above were excised 24 h after administration of the last dose of either curcumin or vehicle alone to measure DNMT1 protein expression. DNMT1 protein was measured via immunoblotting (C). Data for three individual representative mice from each treatment group are shown. Numbers beneath each lane represent quantification of DNMT1 by densitometry, normalized to β-actin. (D) *DNMT1* mRNA expression was assessed via real-time RT-PCR and normalized to *GAPDH* internal control. Values depicted as the percent change were calculated as the change relative to treatment with carrier control. **, *p*<0.01.

To determine whether our *in vitro* and *ex vivo* studies might also have physiological relevance, we corroborated our findings with an *in vivo* study, using an MV4–11 leukemia cell-engrafted animal model in which mice were treated with vehicle control or curcumin by intraperitoneal (IP) injection. Analysis using a linear mixed effect model demonstrated that curcumin significantly inhibited tumor growth compared to mice that received IP injection with vehicle control (*p*<0.0001) (Figure 5A), thus indicating that curcumin may have significant anti-tumor activity in AML. Twenty-eight days after the initicail treatment, we sacificed the mice and compared the weight of tumors generated from each of the two treatment groups. We found that, compared to the vehicle control, curcumin treatment reduced tumor weight by 70% (*p*<0.0001) (Figure 5B). Surprisingly, although curcumin significantly inhibited tumor growth in these mice, we were unable to find any obvious toxicity associated with curcumin treatment (for example, we did not detect a significant loss in body weight of curcumin-treated mice to mice from the vehicle-treated control group). We also measured levels of *DNMT1* protein and transcript expressed by tumors removed from mice treated with curcumin or vehicle control. Consistent with our observations regarding curcumin’s ability to inhibit tumor growth *in vivo* (Figure 4) and down-regulate DNMT1 expression *in vitro* and *ex vivo* (Figure 1), we found that decreased levels of DNMT1 protein and mRNA were expressed by tumor cells isolated from curcumin-treated mice (Figure 5C and 5D).

## Discussion

It has been well-documented that DNMT1, in conjunction with other DNA methyltransferases, regulates and maintains DNA methylation patterns in mammalian cells [28]. Recently, we identified curcumin as a substance which acts as an inhibitor of DNA methyltransferase enzymatic activity and induces significant global DNA hypomethylation in AML cells [19]. In this study, we first demonstrated that curcumin decreases DNMT1 mRNA and protein expression levels, most likely through inhibiting expression of positive regulators of DNMT1, such as Sp1 and the p65 component of NF-κB component, and/or altering their ability to bind to the promoter region of *DNMT1*. Curcumin may serve dual functions: acting as both a chemical inhibitor and a transcriptional modulator of DNMT1. Both of these activities may drive promoter hypomethylation, resulting in reactivation of TSGs. We also found that, in response to curcumin treatment, AML cells underwent apoptosis and became arrested at the G1 phase of the cell cycle. All of these events may occur together, and subsequently contribute to curcumin’s anti-leukemic effects in AML, as evidenced by our *in vitro* and *in vivo* studies.

In contrast to hypomethylators like 5-azanucleosides (e.g. decitabine), which require incorporation into newly synthesized DNA and act to trap DNMT1 protein, curcumin functions as a hypomethylator by perturbing the synthesis of DNMT1 and chemically inhibiting its enzymatic activity. Due to its unique mode of action, curcumin might therefore represent a hypomethylating agent which might be used together with 5-azanucelosides as an additional means to bolster hypomethylating activity. Furthermore, because these drugs operate using two distinct mechanisms, when administered together, they are likely to have additive – if not synergistic – effects in terms of hypomethylation and anti-leukemic activity. This hypothesis is currently being tested in our lab.

There are a number of additional limitations for the use of azanucleosides. In particular, these drugs can cause myelosuppression [29] and are only useful during S phase, when cells are actively dividing; furthermore, azanucleosides are unstable, and can be degraded via hydrolytic cleavage and deamination by cytidine deaminase [30]. Indeed, a variety of conditions may diminish the activity of azanucleosides or even limit their use. In contrast, curcumin represents an alternative hypomethylating agent which may be devoid of several of these limitations, and therefore might serve as a complementary therapy, especially when used in conjunction with azanucleosides or in the setting of azanucleoside-resistance, during treatment of leukemia.

Because our study only investigated curcumin’s role as an epigenetic modulator of methylation, we cannot exclude the possibility that curcumin may also modulate other epigenetic events, such as acetylation, in AML. Indeed, in other cell types, curcumin has been shown to down-regulate histone deacetylases (HDACs) and to inhibit p300/CBP histone acetyltransferase (HAT) activity [31], [32]. Interestingly, curcumin’s ability to suppress p300/CBP HAT activity may be at least partially due to inhibition of NF-κB signaling which, as we demonstrated in this study, is also involved in regulating curcumin’s activity as a DNMT1 modulator. In addition, it is also possible that curcumin’s efficacy in the treatment and/or prevention of cancer may also be due, at least in part, to its ability to modulate expression of microRNAs (miRNAs), which have been shown to be associated with cancer development [33].

The clinical benefits of curcumin as a single agent have been demonstrated in patients with advanced pancreatic cancer [34], and curcumin is currently being tested in a clinical trial for the treatment of multiple myeloma. In this study, we showed that curcumin inhibits growth of AML cells *in vitro* and *in vivo*. The data from our study, along with the aforementioned findings of others, serve as convincing evidence to support the utility of curcumin for the treatment and/or prevention of certain types of cancer(s); furthermore, our current work suggests that curcumin-mediated epigenetic modulation may be at least partly responsible for its efficacy. However, curcumin could not completely suppress the tumor growth, as tumors continue to grow after discontinuation of curcumin treatment. Therefore, as mentioned previously, curcumin therapy may be administered in combination with other clinically effective agents (e.g. decitabine) to treat AML. Therefore, as mentioned previously, ideally curcumin therapy may be administered in combination with other clinically effective agents (e.g. decitabine) to treat AML.

In the current study, we administered free curcumin via i.p. injection to mice bearing xenografts of MV4–11 AML cells. However, the i.p. route of administration, especially when used for a prolonged periods of time, might not represent a realistic treatment strategy in humans. Since maintaining an adequate concentration of curcumin in the plasma seems to be essential for achieving the desired pharmacological effects, the limited bioavailability of free curcumin, when it is administered orally to humans, poses a major clinical obstacle, thereby limiting the potential application of free curcumin as an effective anti-leukemic drug for oral ingestion. Currently, there is considerable interest in developing novel nutraceutical delivery systems to enhance the extent of absorption and bioavailability of curcumin. In particular, a lipid-based nanoemulsifying formulation is emerging technology that has been shown to enhance the oral bioavailability of curcumin [35]. Recently, we successfully optimized a novel nanoemulsifying curcumin (NEC) dosage form that, when compared to suspension curcumin, has been shown to deliver much higher levels of curcumin to the plasma in a murine model [36]. Similar results were observed in a pilot cross-over single dose pharmacokinetic (PK) study using four healthy volunteers in an IRB-approved clinical protocol in which we compared NEC containing 2 grams of curcumin to a standard curcumin C3 complex (CC3C) containing 4 grams of curcumin. In particular, our human data demonstrated that the novel NEC formulation resulted in 200- to 350-fold higher peak plasma levels and 80- to 600-fold higher area under the curve (AUC) of circulating curcumin species, when compared to those results obtained after administering CC3C (data to be reported elsewhere). Notably, no adverse effects were documented in any of the four healthy human subjects involved in our pilot study. Importantly, we also observed in a murine model that treatment with orally administered nanoemulsion curcumin containing 300 mg/kg curcumin (corresponding to clinical dose of 1.8 g for a 60-kg adult) significantly inhibited the growth of cancer (e.g. breast cancer) cell line-engrafted tumors by up to 60% compared to tumors in mice from the non-treatment group (data not shown). Therefore, nanoemulsion curcumin may provide a means of clinically administering orally-ingested curcumin, which may be used alone or together with other clinically effective agents to treat patients with AML. Further testing of orally administered nanoemulsion curcumin is currently underway at our institute.

In summary, here we demonstrate that curcumin down-regulates DNMT1 expression, which results in DNA hypomethylation and reactivation of TSGs in AML, and also correlates to its role as an inhibitor of tumor growth, both *in vitro* and *in vivo*. Importantly, additional studies – to improve *in vivo* bioactivity and/or to investigate the use of curcumin in combination with other therapies – are warranted to determine how best to translate these promising initial findings to preventative and/or clinical treatments.
